# Gut Microbiota Composition Is Related to AD Pathology

**DOI:** 10.3389/fimmu.2021.794519

**Published:** 2022-01-31

**Authors:** Barbara J. H. Verhaar, Heleen M. A. Hendriksen, Francisca A. de Leeuw, Astrid S. Doorduijn, Mardou van Leeuwenstijn, Charlotte E. Teunissen, Frederik Barkhof, Philip Scheltens, Robert Kraaij, Cornelia M. van Duijn, Max Nieuwdorp, Majon Muller, Wiesje M. van der Flier

**Affiliations:** ^1^ Department of Internal Medicine - Geriatrics, Amsterdam Cardiovascular Sciences, Amsterdam University Medical Center (UMC), Amsterdam, Netherlands; ^2^ Department of Internal and Vascular Medicine, Amsterdam University Medical Center (UMC), Amsterdam, Netherlands; ^3^ Alzheimer Center, Department of Neurology, Amsterdam Neuroscience, Amsterdam University Medical Center (UMC), Amsterdam, Netherlands; ^4^ Department of Clinical Chemistry, Amsterdam University Medical Center (UMC), Amsterdam, Netherlands; ^5^ Department of Radiology and Nuclear Medicine, Amsterdam University Medical Center (UMC), Amsterdam, Netherlands; ^6^ University College London (UCL) Institutes of Neurology, Faculty of Brain Sciences, London, United Kingdom; ^7^ Department of Internal Medicine, Erasmus Medical Center (MC), Rotterdam, Netherlands; ^8^ Department of Epidemiology, Erasmus Medical Center (MC), Rotterdam, Netherlands; ^9^ Nuffield Department of Population Health, University of Oxford, Oxford, United Kingdom; ^10^ Department of Epidemiology and Data Science, Amsterdam University Medical Center (UMC), Vrije Universiteit Amsterdam, Amsterdam, Netherlands

**Keywords:** gut microbiota, microbiome, Alzheimer’s disease, amyloid beta, P-tau, MRI

## Abstract

**Introduction:**

Several studies have reported alterations in gut microbiota composition of Alzheimer’s disease (AD) patients. However, the observed differences are not consistent across studies. We aimed to investigate associations between gut microbiota composition and AD biomarkers using machine learning models in patients with AD dementia, mild cognitive impairment (MCI) and subjective cognitive decline (SCD).

**Materials and Methods:**

We included 170 patients from the Amsterdam Dementia Cohort, comprising 33 with AD dementia (66 ± 8 years, 46%F, mini-mental state examination (MMSE) 21[19-24]), 21 with MCI (64 ± 8 years, 43%F, MMSE 27[25-29]) and 116 with SCD (62 ± 8 years, 44%F, MMSE 29[28-30]). Fecal samples were collected and gut microbiome composition was determined using 16S rRNA sequencing. Biomarkers of AD included cerebrospinal fluid (CSF) amyloid-beta 1-42 (amyloid) and phosphorylated tau (p-tau), and MRI visual scores (medial temporal atrophy, global cortical atrophy, white matter hyperintensities). Associations between gut microbiota composition and dichotomized AD biomarkers were assessed with machine learning classification models. The two models with the highest area under the curve (AUC) were selected for logistic regression, to assess associations between the 20 best predicting microbes and the outcome measures from these machine learning models while adjusting for age, sex, BMI, diabetes, medication use, and MMSE.

**Results:**

The machine learning prediction for amyloid and p-tau from microbiota composition performed best with AUCs of 0.64 and 0.63. Highest ranked microbes included several short chain fatty acid (SCFA)-producing species. Higher abundance of *[Clostridium] leptum* and lower abundance of *[Eubacterium] ventriosum* group spp., *Lachnospiraceae* spp., *Marvinbryantia* spp., *Monoglobus* spp., *[Ruminococcus] torques group* spp., *Roseburia hominis*, and *Christensenellaceae R-7* spp., was associated with higher odds of amyloid positivity. We found associations between lower abundance of *Lachnospiraceae* spp., *Lachnoclostridium* spp., *Roseburia hominis* and *Bilophila wadsworthia* and higher odds of positive p-tau status.

**Conclusions:**

Gut microbiota composition was associated with amyloid and p-tau status. We extend on recent studies that observed associations between SCFA levels and AD CSF biomarkers by showing that lower abundances of SCFA-producing microbes were associated with higher odds of positive amyloid and p-tau status.

## Introduction

Alzheimer’s disease (AD) is the most common cause of dementia, and is characterized by the accumulation of amyloid beta in plaques and the formation of neurofibrillary tangles including hyperphosphorylated tau (p-tau). Another hallmark is chronic neuroinflammation, which is reflected by activation of microglia and increased cytokine production (1). The gut microbiome has been shown to interact with the innate and adaptive immune system, by release of bacterial toxins and production of metabolites (2, 3). As has been shown in other neurological conditions such as multiple sclerosis (4, 5), gut microbiota could affect neuroinflammation.

The gut is populated with trillions of microbiota, including bacteria, viruses, fungi, archaea and protozoa (6). Collectively, the genomes of these cells are referred to as the gut microbiome. The microbiota composition is affected by dietary factors, age, sex, body mass index (BMI) and medication use, including antibiotics, metformin, proton pump inhibitors and statins (7). Gut microbiota live in symbiosis with the host and are needed for the degradation of macronutrients and production of metabolites (8, 9). Short chain fatty acids (SCFAs) are key metabolites of the gut microbiota, which are produced by fermentation of indigestible dietary fibers (10).

Animal studies have reported differences in gut microbiota composition between AD and wild-type mice, including a decrease in SCFA-producing microbes (11, 12). Fecal microbiota transplantation from wild type mice to AD-like animal models such as APP/PS1 and ADLP^APT^ mice resulted in a reduction of amyloid, suggesting a causal relation between gut microbes and AD (12, 13). Colonization of Tg2576 mice with *Bacteroides* exacerbates amyloid depositions, suggesting a mechanism for the impact of gut microbiota on AD pathology (14). In addition, an intervention with sodium butyrate, an SCFA, in an AD mice model resulted in a reduction of AD pathology (15).

In line with these animal studies, five human studies observed alterations in microbiota composition in patients with AD or mild cognitive impairment (MCI) compared to controls, with a lower abundance of SCFA-producing species in patients with AD (16–20). However, the nature of the specific microbiota alterations was conflicting across studies, with for instance lower (16, 19, 20) and higher (17) abundance of *Ruminococcaceae* spp., and lower (17) and higher (16, 18, 19) abundance of the *Bacteroidetes* phylum of MCI or AD patients compared to controls. In addition, former studies did not take into account AD pathology as measured with AD biomarkers (17–20), while studies that did focused on a limited set of microbes for these analyses (16, 21).

Hence, we aimed to assess the relation between gut microbiota composition, as measured with 16S rRNA sequencing, and biomarkers of AD pathology, including CSF biomarkers and MRI measures of vascular burden and neurodegeneration, in a memory clinic population with AD dementia, mild cognitive impairment (MCI) and subjective cognitive decline (SCD).

## Methods

### Study Population

**Figure 1:**
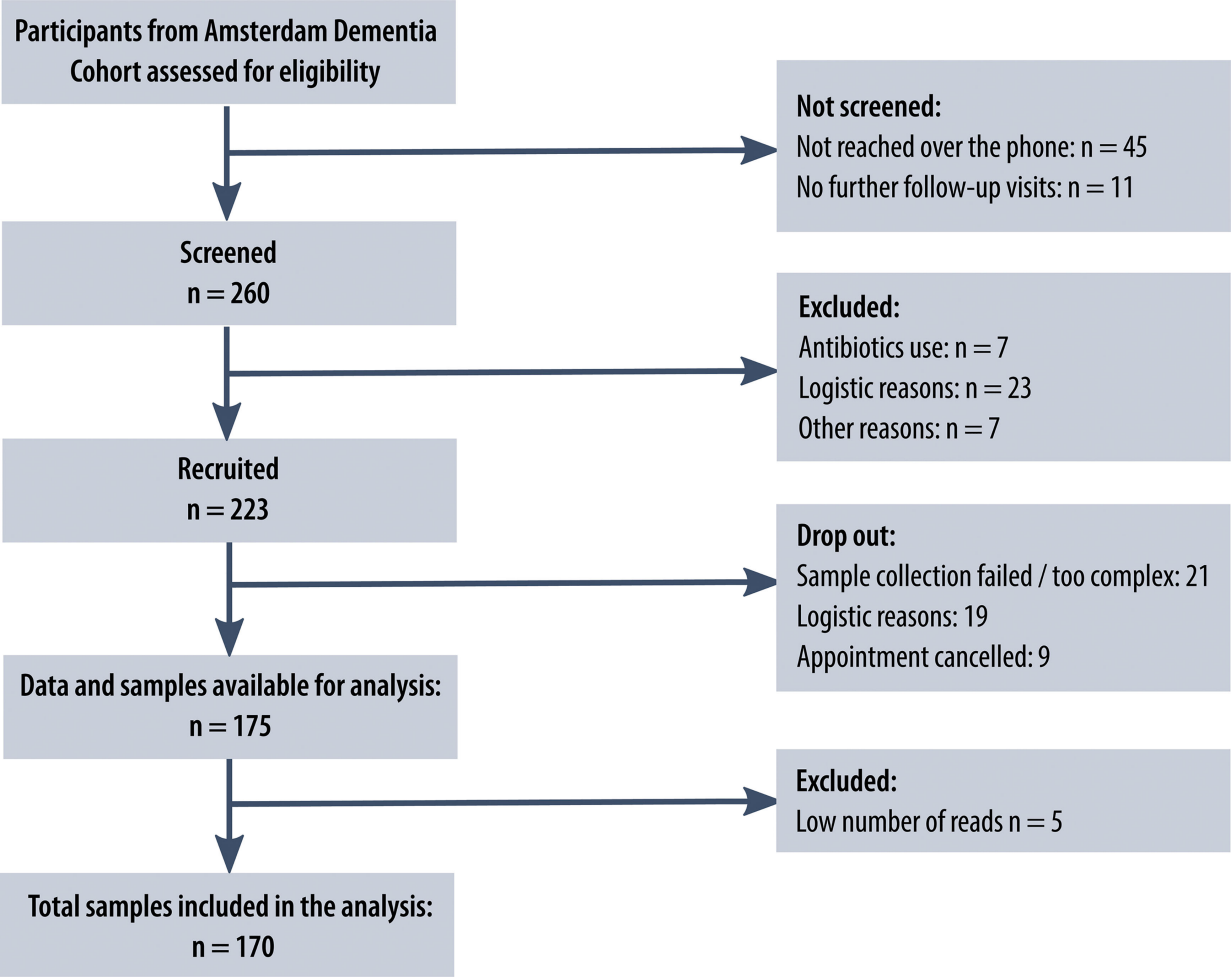
Study flowchart. Flowchart of the number of patients from the Amsterdam Dementia Cohort screened, recruited and included in the analysis, including reasons for exclusion at different stages. The flowchart was designed following the ‘Strengthening The Organization and Reporting of Microbiome Studies’ (STORMS) checklist (22).

We invited 223 study participants from the Amsterdam Dementia Cohort and SCIENCe project, for fecal sample collection. All invited participants were diagnosed with AD dementia, MCI or SCD and had mini-mental state examination (MMSE) scores higher than 16. Of the invited participants, 175 subjects collected samples, and 170 subjects could be included in our analyses (**Figure 1**), comprising 33 patients with AD, 22 patients with MCI and 120 subjects with SCD (23–25). All patients underwent comprehensive neuropsychological assessment, neurological examination, lumbar puncture and MRI as part of a standard dementia screening (23). MCI and AD diagnoses were established by consensus in a multidisciplinary meeting according to the National Institute on Aging-Alzheimer’s Association criteria (26, 27). Subjects with SCD presented with memory complaints but performed normal on cognitive examinations and did not fulfill criteria for MCI, dementia, psychiatric diagnoses or other neurological diagnoses (23). Patients were seen annually for follow-up visits, during which cognitive assessments and medical examinations were repeated. Prior to these follow-up visits, patients were asked to collect fecal samples. The study protocol was approved by the Ethics Committee of the Amsterdam UMC, and all study participants provided written informed consent.

Descriptive characteristics included age, sex, medical history (history of hypertension, hypercholesterolemia and diabetes; self-reported or described in a referral letter), medication use [antihypertensive medication, glucose lowering medication, cholesterol lowering medication, proton pump inhibitors (PPI)], smoking status (current smoking yes/no) and alcohol use (in units per day). Global cognitive functioning was assessed using the MMSE (scale 0-30) (28).

### Gut Microbiota Composition

Patients were sent a fecal collection kit prior to their memory clinic follow-up visit. Seven patients who used antibiotics within three months prior to collection were not included. Other exclusion criteria were diarrhea in the past week or severe gastro-intestinal conditions, including inflammatory bowel disease. A flowchart with the screening and recruiting procedure and reasons for exclusion at each stage is presented in **Figure 1**. The included patients were asked to store the sample in a freezer and to transport the samples to the hospital in a cooling bag. The 175 samples were shipped to Erasmus Medical Center, Rotterdam, the Netherlands, for sequencing. Aliquots of ~300 mg feces were homogenized and DNA was isolated using bead-beating and the InviMag Stool DNA kit (Invitek Molecular GmbH, Berlin, Germany) on a KingFisher Flex robot (Thermo Fisher Scientific, Breda, Netherlands). Fecal microbiota composition was determined by sequencing the V3 and V4 hypervariable regions of the 16S rRNA gene on an Illumina MiSeq platform (Illumina Inc., San Diego, CA, USA) using 319F (ACTCCTACGGGAGGCAGCAG) −806R (GGACTACHVGGGTWTCTAAT) primers and dual-indexing (29). The processing of the raw sequencing data is described in **Supplement 1**, which after rarefying to 20.000 counts per sample resulted in a dataset with 170 samples and 7894 amplicon sequence variants (ASVs). Prior to the machine learning analyses, we filtered for ASVs that had at least 5 counts in 30% of the subjects, which resulted in a dataset with 181 ASVs. Of these ASVs, taxonomy was available up to species level for 32%, up to genus level for 88% and up to family level for 99%.

### AD Biomarkers

CSF was obtained by lumbar puncture using a 25-gauge needle and collected in 10 ml polypropylene tubes (Sarstedt). Amyloid-β_1-42_ (Aβ_42_) and p-tau concentrations were determined with sandwich ELISAs, using Innotest (Fujirebio) and Elecsys immunoassays. Patients were classified as having a positive amyloid status, indicative for AD pathology, if they had amyloid values lower than the platform-dependent cut-off (Innotest <813 pg/ml (30, 31); Elecsys <1000 pg/ml). A positive p-tau status was defined as having p-tau values higher than the platform-dependent cut-off (Innotest >52 pg/ml; Elecsys >19pg/ml). Because of the high correlations between these platforms, Elecsys values were converted to Innotest values (32). CSF biomarkers were available for 116 patients at a median of 2.4 [IQR 2.2, 3.2] years before the time of fecal sampling.

MRI scans were performed on a 3.0T scanner and the protocol included T1-weighted, T2-weighted, fluid-attenuated inversion recovery (FLAIR) and gradient echo T2*-weighted images. A trained neuroradiologist evaluated all scans using visual rating scales. Medial temporal atrophy (MTA) was rated on coronal reconstructions of T1-weighted images of both sides, perpendicular to the long axis of the hippocampus (0-4 scale). MTA was averaged across left and right scores, and was dichotomized with a cut-off of ≥1 (33, 34). Global cortical atrophy (GCA) was assessed on transverse FLAIR images and rated using a 4-point scale (0-3) and dichotomized (cut-off ≥1) (34, 35). White matter hyperintensities (WMH) were assessed on the same sequences using the Fazekas scale for white matter hyperintensities (0-3) and dichotomized with a cut-off of ≥2 (36). Microbleeds were defined as oval or round hypointense lesions up to 10 mm on a T2*-weighted MRI. Microbleeds counts were dichotomized into present or absent (37). MRI results were available for 136 patients at a median of 2.1 [IQR 0.5, 2.4] years before the time of fecal sampling.

### Statistical Analysis

Differences in descriptive and outcome variables between diagnosis groups were tested using analysis of variance for continuous variables with normal distributions, Kruskal-Wallis tests for continuous variables with non-normal distributions and chi-square tests for categorical variables. To compare microbiota composition between groups, we calculated alpha diversity indices, including Shannon index, richness and Faith’s phylogenetic diversity (38, 39). In addition, we compared beta diversity between groups by testing differences in Bray-Curtis distance with a PERMANOVA test. We used the rarefied microbiota data to calculate alpha and beta diversity.

We used machine learning models to predict dichotomized AD biomarkers, including amyloid and p-tau status, MTA, GCA, WMH and microbleeds, from gut microbiota composition (i.e. the relative abundance of ASVs). Subjects were excluded for a particular model if data on that outcome variable were missing. Microbiota abundance data is compositional data, with skewed, zero-inflated and overdispersed distributions. We used gradient-boosted tree models [XGBoost algorithm (40)], which is a state-of-the art algorithm that has shown good accuracy in comparative microbiota studies (41). To prevent overfitting, we used a nested cross-validation design in performing these models (**Supplement 2**). In each of the 200 iterations, the dataset was randomly split into a test set containing 20% of the subjects and a training set with the remaining 80%. Within the train set, 5-fold cross-validation was performed in order to optimize the model hyperparameters. Two random variables were added to the microbiota data in each iteration as a benchmark. The resulting model was evaluated on the test set which yielded an area under the receiver-operator curve (AUC) as main model quality metric, and a ranked list of microbial predictors with their relative importance to the model. These were recorded for each iteration and were averaged across 200 iterations.

We selected the two machine learning models with the highest AUCs for logistic regression, to obtain effect sizes for the associations between the 20 highest ranked (i.e. highest feature importance) microbes and the dichotomous outcome of these machine learning models. We ran three models: model 1 adjusted for age, sex and BMI, model 2 with additional adjustment for diabetes, statin and proton pump inhibitor (PPI) use and model 3 with additional adjustment for MMSE. The effect sizes, reported as odds ratios (OR) per log2-increase in counts with 95%-confidence intervals (95%-CI) were visualized in a forest plot. Spearman rank correlation coefficients were calculated between the top 10 best predicting ASVs found by the two best performing machine learning models and the AD biomarkers and were visualized with a correlation heatmap. We used hierarchical clustering (Ward’s method) to order the ASVs in this plot and to draw a dendrogram. The correlations with amyloid levels and MMSE scores were inversed for interpretability, since lower levels are indicative for AD pathology in contrast to other biomarkers.

Machine learning was implemented in Python (v. 3.7.4) using the XGBoost (v. 0.90), numpy (v. 1.16.4), pandas (v. 0.25.1), and scikit-learn (v. 0.21.2) packages. Statistical analyses and visualizations were performed using R version 3.6.2.

### Data Availability

The sequencing data presented in this study can be found in an online repository, European Nucleotide Archive (ENA) accession number PRJEB49329 (https://www.ebi.ac.uk/ena/browser/view/PRJEB49329). Clinical data are available upon reasonable request at Alzheimer Center Amsterdam, Amsterdam UMC, location VUmc in Amsterdam, The Netherlands.

## Results

### Population Characteristics

**Figure 2:**
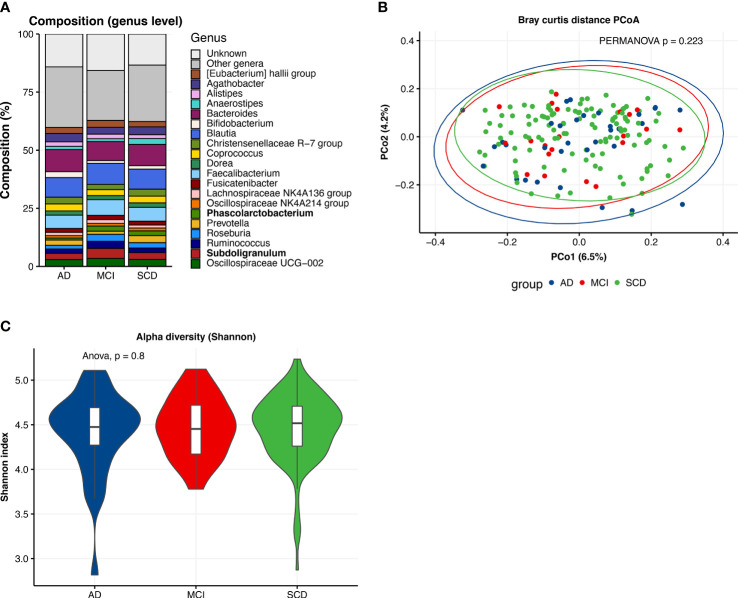
Descriptive characteristics of microbiota composition, differences between diagnosis groups. **(A)** Compositional plot of top 20 genera with bars representing diagnosis groups: Alzheimer’s disease dementia (AD), mild cognitive impairment (MCI) and subjective cognitive decline (SCD). “Unknown” refers to ASVs of which taxonomy was not known up to genus level. Genera with different abundances across groups (Kruskal-Wallis test, p <0.05) are marked in bold. **(B)** Principal coordinate analysis (PCoA) plot of Bray-Curtis distances per diagnosis group with PERMANOVA test for group differences. **(C)** Alpha diversity (Shannon index) of gut microbiota composition per diagnosis group.

The mean age of the overall study population was 63 years (**Table 1**), with the AD dementia group (66.0±8.0) older than the SCD group (62.0±7.5; p<0.05). Patients with AD dementia, MCI and SCD were comparable in terms of sex, BMI, smoking status and alcohol use, as well as most cardiovascular risk factors. However, diabetes was more prevalent among patients with AD dementia and MCI compared to SCD (p<0.05). AD dementia and MCI patients more often had abnormal AD biomarkers than controls, such as positive amyloid and p-tau status (p<0.001), and MTA (p<0.01) and GCA scores ≥1 (p<0.05). Distributions of amyloid and p-tau CSF levels are presented in **Supplement 3**. Prevalence of WMH ≥2 and microbleeds tended to be higher in patients with MCI, but this difference was not significant. The gut microbiota composition on genus level of the three diagnosis groups is shown in **Figure 2**. When comparing the 20 most abundant genera between diagnosis groups, only two genera, *Subdoligranulum* (p<0.05) and *Phascolarctobacterium* (p<0.05), had different abundances between groups. There were no differences in beta diversity (PERMANOVA p=0.223), nor in alpha diversity, as measured with Shannon index, richness and Faith’s phylogenetic diversity.

**Table 1:** Patient characteristics.

	N	Overall	AD dementia	MCI	SCD	p
		170	33	21	116	
Age	170	63.1±7.8	66.0±8.0^a^	64.1±7.9	62.0±7.5	**0.028**
Female sex	170	75 (44.1)	15 (45.5)	9 (42.9)	51 (44.0)	0.981
BMI	144	25.3±4.0	25.2±3.7	24.0±3.3	25.6±4.1	0.289
Current smoking	129	12 (9.3)	0 (0.0)	2 (11.8)	10 (10.6)	0.338
Alcohol units/day	130	1.3±1.5	1.2±1.4	1.3±1.3	1.3±1.5	0.908
Hypertension	170	42 (24.7)	12 (36.4)	4 (19.0)	26 (22.4)	0.212
Diabetes	170	15 (8.8)	5 (15.2)	4 (19.0)	6 (5.2)	**0.043**
Hypercholesterolemia	170	29 (17.1)	5 (15.2)	5 (23.8)	19 (16.4)	0.671
Antihypertensive drugs	170	55 (32.4)	13 (39.4)	5 (23.8)	37 (31.9)	0.482
Cholesterol lowering drugs	170	48 (28.2)	11 (33.3)	6 (28.6)	31 (26.7)	0.758
Glucose lowering drugs	170	12 (7.1)	4 (12.1)	3 (14.3)	5 (4.3)	0.117
Proton pump inhibitors	170	29 (17.1)	6 (18.2)	2 (9.5)	21 (18.1)	0.618
MMSE	161	29 [26, 30]	21 [19, 24]^a,b^	27 [25, 29]^a^	29 [28, 30]	**<0.001**
ApoE4 allele	166	74 (44.6)	24 (75.0)^a^	12 (57.1)	38 (33.6)	**<0.001**
amyloid positive status	115	49 (42.6)	24 (96.0)^a,b^	8 (47.1)	17 (23.3)	**<0.001**
amyloid CSF levels	115	884 [646-1100]	589 [526-663]^a,b^	875 [643-943]^a^	1034 [828-1188]	**<0.001**
p-tau positive status	116	71 (61.2)	26 (100.0)^a^	14 (82.4)^a^	31 (42.5)	**<0.001**
p-tau CSF levels	116	56 [45-88]	100 [80-140]^a,b^	78 [54-107]^a^	49 [34-58]	**<0.001**
MTA≥1	137	41 (29.9)	12 (54.5)^a^	7 (41.2)	22 (22.4)	**0.007**
GCA≥1	137	49 (35.8)	11 (50.0)	10 (58.8)^a^	28 (28.6)	**0.018**
WMH≥2	137	15 (10.9)	2 (9.1)	3 (17.6)	10 (10.2)	0.633
Microbleeds present	137	24 (17.5)	4 (18.2)	6 (35.3)	14 (14.3)	0.109

Patient characteristics are presented as mean ± SD, median [interquartile range] or n (%). Differences were tested with one-way ANOVA for continuous variables with normal distribution, and Kruskal-Wallis test for continuous variables with non-normal distribution, or chi-square tests for categorical variables. ^a^Significantly different from SCD upon post-hoc testing, ^b^Significantly different from MCI upon post-hoc testing. CSF, cerebrospinal fluid; MTA, medial temporal atrophy; GCA, global cortical atrophy; WMH, white matter hyperintensities.

Significant p-values (p < 0.05) are marked in bold.

### Associations Gut Microbiota Composition and AD Biomarkers

**Figure 3:**
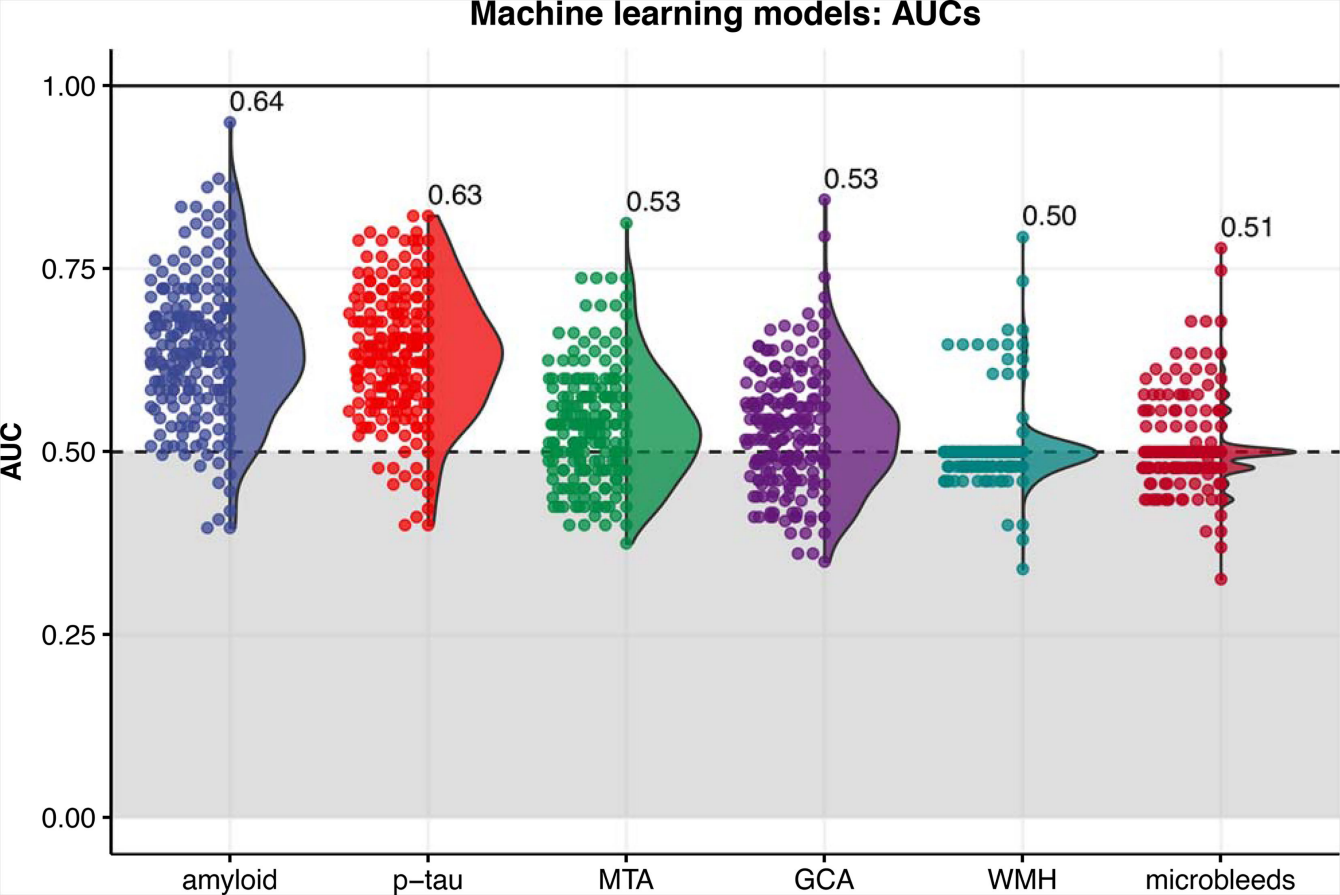
Distribution of area under the receiver-operating curves (AUCs) resulting from 200 iterations of the machine learning classification models (XGBoost algorithm) for each outcome. The labels indicate the mean AUC over 200 iterations. MTA, medial temporal atrophy; GCA, global cortical atrophy; WMH, white matter hyperintensities.

The machine learning model for the prediction of amyloid status from gut microbiota composition performed best with an AUC of 0.64±0.10 (**Figure 3**). This model was closely followed by the p-tau model with an AUC of 0.63±0.09, while AUCs of the MRI visual scores ranged between 0.50 and 0.53. Highest ranked predictors of the amyloid (CSF) predicting model with all subjects included *[Eubacterium] ventriosum* group spp., *Subdoligranulum* spp., and *Anaerostipes* spp. In the model predicting p-tau, highest ranked microbes included *Lachnospiraceae* spp., *Lachnoclostridium edouardi* and *Blautia faecis*. These microbes are all anaerobic bacteria from the *Firmicutes* phylum and *Eubacterieae, Ruminococcaceae* and *Lachnospiraceae* families that are known for production of SCFAs. Some ASVs, including *Subdoligranulum* spp., *Roseburia hominis* and *Butyricoccus* spp., could be found in the top 20 predictors of both the amyloid and p-tau model. The receiver-operating curves (ROCs) of the amyloid and p-tau models with the relative importance of the highest ranked predictors can be found in **Supplement 4**.

**Figure 4:**
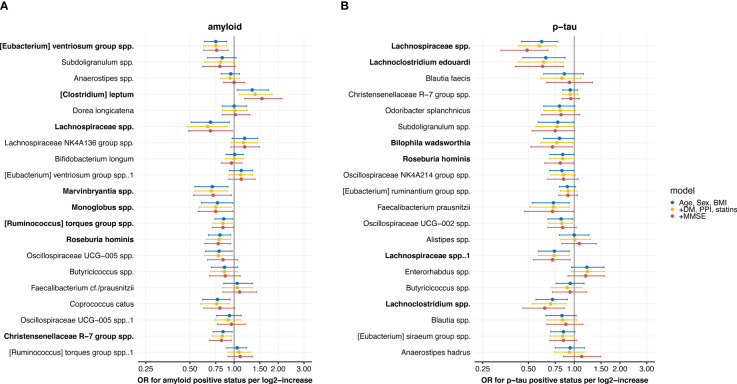
Forest plots with results from the logistic regression models with associations between the 20 highest ranked microbial predictors from the machine learning model, ordered by ranking, and **(A)** amyloid and **(B)** p-tau positive status. Three models are shown: 1) adjusted for age, sex and body mass index (BMI), 2) additionally adjusted for diabetes mellitus (DM), use of proton pump inhibitors (PPI) and statins and 3) additionally adjusted for mini-mental state examination (MMSE) score. Results are presented as odds ratios (OR) with 95% confidence intervals. Microbes with significant associations in the fully adjusted model are marked in bold.

Logistic regression models showed significant associations with amyloid status for 10 of the 20 highest ranked microbial predictors from the amyloid status machine learning model (**Figure 4A**) in model 1 and 2. Two ASVs, *Coprococcus catus* (OR 0.78 (0.63-0.97), p<0.05; model 2) and *Oscillospiraceae UCG-005* spp. (OR 0.76 (0.59-0.93), p<0.05; model 2), were only associated with amyloid status in model 1 and 2. Eight associations remained significant in model 3, adjusting for age, sex, BMI, diabetes, proton pump inhibitor and statin use, and MMSE, including *[Eubacterium] ventriosum* group spp. (OR 0.76 (0.62-0.91) per log2-increase in counts, p<0.01), *Lachnospiraceae* spp. (OR 0.69 (0.49-0.97), p<0.05), *Marvinbryantia* spp. (OR 0.72 (0.53-0.96), p<0.05), *Monoglobus* spp. (OR 0.75 (0.57-0.98)), *[Ruminococcus] torques group* spp. (OR 0.84 (0.71-0.99), p<0.05), *Roseburia hominis* (OR 0.78 (0.63-0.95), p<0.05), and *Christensenellaceae R-7* spp. (OR 0.82 (0.68-0.96), p<0.05), and *[Clostridium] leptum* spp. (OR 1.55 (1.18-2.12), p<0.01).

Six of the top 20 highest ranked microbial predictors from the p-tau status model were associated with p-tau status in the fully adjusted model 3 (**Figure 4B**). These included two *Lachnospiraceae* spp. ASVs (OR 0.49 (0.33-0.67), p<0.001, and OR 0.72 (0.54-0.94), p<0.05), *Lachnospiraceae edouardii* (OR 0.62 (0.41-0.85), p<0.01) and *Lachnoclostridium* spp. (OR 0.72 (0.54-0.94), p<0.01), which all belong to the *Lachnospiraceae* family. In addition, *Roseburia hominis* (OR 0.81 (0.64-0.99), p<0.05) and *Bilophila wadsworthia* (OR 0.72 (0.52-0.97), p<0.05) were lower abundant in patients with a positive p-tau status.

### Associations of Top Predicting Microbes With Other Biomarkers

**Figure 5:**
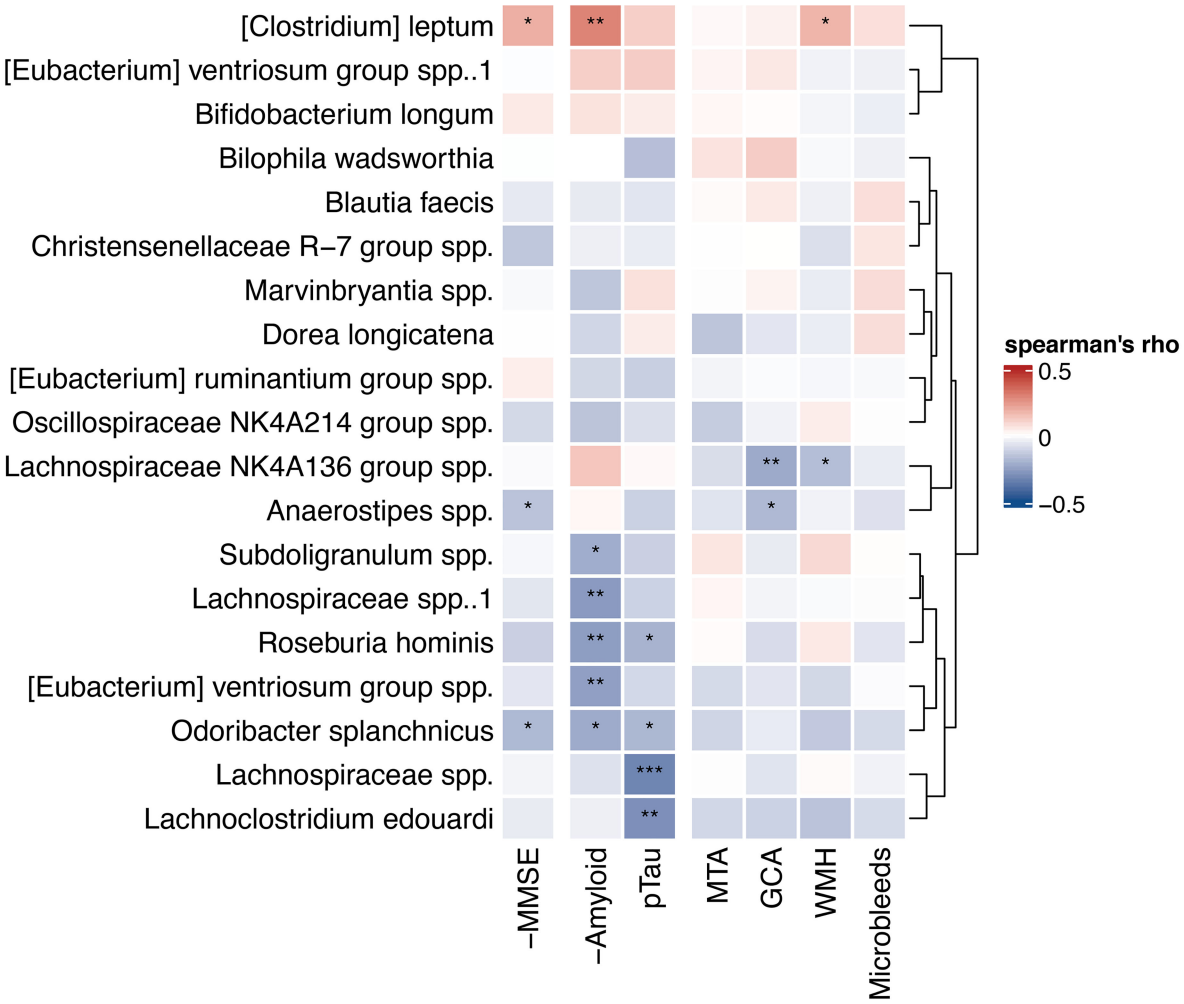
Heatmap of correlations with highest ranked predictors. Spearman’s correlations between 10 highest ranked microbial predictors from the amyloid and p-tau machine learning models and continuous AD biomarkers. Hierarchical clustering (Ward’s method) was used to order the microbes and draw the dendrogram on the right. Correlations with MMSE and amyloid CSF levels are reversed for interpretability (-MMSE and -Amyloid), as lower values of these variables are indicative for pathology, in contrast to the other biomarkers. Negative (blue) correlations in this heatmap reflect correlations with less biomarkers indicative for AD pathology. *p < 0.05, **p < 0.01, ***p < 0.001. MMSE, mini-mental state examination; P-tau, phosphorylated tau; MTA, medial temporal atrophy; GCA, global cortical atrophy; WMH, white matter hyperintensities.

We also calculated Spearman’s correlations between the 10 highest ranked microbes from the amyloid and p-tau models (19 microbes in total, because of an overlap of one ASV) and all AD biomarkers, including amyloid and p-tau levels (**Figure 5**). Five ASVs correlated with higher amyloid levels (0.27<r_s_<0.22), while one ASV, *[Clostridium] leptum*, correlated with lower amyloid levels (r_s_ 0.29, p<0.01). Four ASVs correlated with lower p-tau levels (-0.33<r_s_<-0.19). *Roseburia hominis* and *Odoribacter splanchicus* correlated with both higher amyloid and lower p-tau levels. *Lachnospiraceae NK4A136 group* spp. and *Anaerostipes* spp. correlated with lower GCA visual scores on MRI. In addition, *Anaerostipes* spp. and *Odoribacter splanchicus* correlated with higher MMSE scores, while *[Clostridium] leptum* correlated with lower MMSE scores.

## Discussion

Our main findings are the associations between gut microbiota composition and CSF amyloid and p-tau status. Discriminative value of the models predicting amyloid and p-tau status from gut microbiota composition was modest, but nonetheless we provide evidence that several SCFA-producing microbes are altered in patients with abnormal CSF amyloid and/or p-tau. We extend on animal studies reporting associations between SCFAs and amyloid pathology by showing that lower abundance SCFA-producing microbes was associated with lower odds of amyloid and p-tau positive status (15, 42).

Five cross-sectional studies of differences in gut microbiota between patients with AD and controls found that several microbes were less abundant in AD, including *Faecalibacterium prausnitzii, Eubacterium*, *Anaerostipes*, *Ruminococcus*, and *Roseburia* spp, while other microbes, such as *Odoribacter splanchicus*, *Bacteroides, Prevotella*, and *Alistipes* spp., were more abundant (16–20). In line with these studies, we found that many of the highest ranked predictors for amyloid and p-tau status belonged to the *Lachnospiraceae* family, including *Roseburia hominis*, *[Ruminococcus] torques, Lachnoclostridium, Monoglobus* and *Marvinbryantia* spp. In contrast to earlier findings, higher abundance of *Odoribacter splanchicus* and *Alistipes* spp. correlated with more normal levels of AD biomarkers (higher amyloid and lower p-tau CSF levels) in our analyses, albeit these associations were lost after adjustment for covariates.

Two previous studies investigated associations between AD biomarkers and a specific subset of gut microbes (16, 21). One cross-sectional study correlated 13 microbial genera, that were differently abundant between AD patients and controls including a few that are SCFA-producing, with amyloid and p-tau levels in 40 patients. *Blautia* and *Bacteroides* spp. were associated with higher levels of biomarkers indicative of AD pathology, while *SMB53* and *cc115* spp. were associated with lower AD biomarkers. Of these genera, only *Blautia faecis* was also among the best predictors for p-tau status in our analyses, although this association was not significant in the adjusted analyses. These different findings could be explained by the older study population or by their inclusion of very low abundance taxa in the statistical analyses. A study that assessed differences between amyloid positive and negative patients in six microbes measured using qPCR found that *Escherichia/Shigella* spp. were more abundant while *Eubacterium rectale* was less abundant in amyloid positive patients (21). Indeed, several *Eubacterium* species were among the highest ranked predictors for amyloid status in our analyses. We did, however, not confirm the *Escherichia/Shigella* association, most likely because qPCR is more sensitive in finding changes in low abundant pathogens than 16S rRNA gene amplicon sequencing. *[Clostridium] leptum*, a microbe from the *Oscillospiraceae* family, was the only ASV associated with higher odds of amyloid positive status, and also correlated with lower continuous amyloid CSF levels. To our knowledge, we are the first to report an association between this microbe and AD biomarkers.

Our analyses allowed us to differentiate between predictors for amyloid and p-tau status. Microbial predictors for amyloid and p-tau status showed some overlap, such as *Roseburia hominis* and *Lachnospiraceae* spp. We also found differences in highest ranked predictors for amyloid compared to p-tau status; microbial strains from the *Eubacterium* and *Ruminococcus* genera were the highest ranked predictors for amyloid status, while several *Lachnoclostridium* spp. were among the highest predictors for p-tau status.

In contrast to our findings in CSF amyloid and p-tau, we did not find associations between microbiota composition and MRI measures including vascular markers such as WMH and microbleeds in our machine learning model (AUC 0.50), perhaps due to the low prevalence of cerebrovascular damage in this young study population. The low prevalence of cerebrovascular damage also makes it unlikely that the observed associations with amyloid and p-tau were mediated by vascular pathology.

There are several hypotheses regarding the mechanisms by which gut microbiota could affect AD pathology which involve several metabolites and toxins. Lipopolysaccharide (LPS) can be found in the outer membrane of gram-negative bacteria and has been shown to elicit peripheral inflammatory responses, affect the permeability of the blood-brain barrier and induce neuroinflammation (43, 44). In contrast, capsular polysaccharide A (PSA) of *Bacteroides fragilis* species has been shown to have anti-inflammatory effects on the peripheral immune system (45), and to suppress central neuroinflammation by induction of T-regulatory cells in mice (46). However, *Bacteroides fragilis* was not among the highest ranked predictors for amyloid nor p-tau status in our analyses, nor were other species from the gram-negative *Bacteroides* genus.

The highest ranked predictors were mostly species from the predominantly gram-positive Firmicutes phylum known for SCFA production. SCFAs, including acetate, propionate and butyrate, are produced by gut bacteria in fermentation processes of otherwise undigestible dietary fibers and have immunomodulatory potential (10, 47). SCFAs could have indirect effects on AD pathology by induction of peripheral inflammation or by altering the integrity of the blood-brain barrier, as shown by a butyrate intervention study in germ-free mice (42). Alternatively, SCFA could have direct anti-inflammatory effects on microglia as was shown in an *in vitro* study (48). In that regard, future studies could focus on associations between fecal and plasma SCFA levels and inflammatory brain markers such as glial fibrillary acidic protein (GFAP) (49).

There are several limitations of our study including the cross-sectional design which warrants caution that observed associations should not be interpreted as causal relationships. Moreover, time lags between the biomarker measurements and the fecal sampling might have confounded some associations. Although we adjusted for relevant confounders such as age, sex, BMI, diabetes and medication use, we cannot rule out residual confounding. Dietary factors in particular have been shown to affect microbiota composition (50). Since AD patients tend to lose weight over the course of the disease, it has been suggested that cognitive decline could lead to lower energy intake which might also affect microbiota composition (51). However, we have found previously that macronutrient intake was not different between diagnosis groups in this cohort (52). Moreover, associations between gut microbiota composition and AD biomarkers remained significant when adjusting for cognitive function (MMSE). Of note, higher abundance of SCFA-producing microbes is indicative for, but does not necessarily reflect higher gut or plasma SCFA levels. To assess microbial production of SCFAs, metagenomic sequencing would be needed, which was not within the scope of the current study.

Strengths of this study include the availability of several AD biomarkers, including CSF and MRI data, and the inclusion of patients in different stages of the AD disease continuum. Fecal samples were obtained using a standardized protocol, participants taking antibiotics were excluded, and microbiota composition was determined with 16S gene amplicon sequencing, which is a widely used sequencing method. Machine learning prediction models enabled us to simultaneously include all ASVs as features in order to find the best predicting microbes. Nested cross-validation ensured robustness of the models and prevented overfitting.

The putative relation between gut microbiota composition and AD pathology, may provide opportunities for future treatment. Different treatment strategies based on modulating gut microbiota composition have been investigated in other diseases such as inflammatory bowel disease and diabetes (53–55). Fecal microbiota transplantation (FMT) aims to restore gut microbiota composition by administering microbiota from healthy donors to diseased subjects through a nasoduodenal tube (55). In obese subjects, FMT has been shown to alter brain dopamine transporter binding, thus pointing towards a gut-brain connection (56). Nonetheless, FMT is logistically challenging and the effects of transplantation fade over time (57, 58). Another strategy includes the use of prebiotics (often fiber supplements) aimed to promote the growth of certain microbes, or probiotics, supplements of beneficial strains (59). A meta-analysis showed positive effects on cognition of *Bifidobacterium* and *Lactobacillus* probiotics in patients with MCI (60). However, beneficial butyrate-producing species are often strictly anaerobic or oxygen sensitive, complicating culturing and probiotic production (61). A third strategy is to directly target microbial pathways such as SCFA production, by interventions with high fiber intake or by administering SCFAs including acetate or sodium butyrate (62, 63).

Concluding, we found associations between gut microbiota composition and AD pathology in our memory clinic cohort. Lower abundance of SCFA-producing microbes was associated with higher odds of AD pathology. SCFAs are known to have peripheral immunomodulatory potential, providing a putative target for treatment.

## Data Availability Statement

The datasets presented in this study can be found in online repositories. The names of the repository/repositories and accession number(s) can be found below: https://www.ebi.ac.uk/ena/browser/home, accession ID: PRJEB49329.

## Ethics Statement

The studies involving human participants were reviewed and approved by Medisch Ethische Toetsingscommissie VUmc. The patients/participants provided their written informed consent to participate in this study.

## Author Contributions

WF, CT, FB, PS, and CD contributed to conception and design of the study. HH, FL, AD, ML, and BV collected the data. RK was responsible for the sequencing of the samples. BV performed the statistical analyses. WF, MN, and MM contributed to the interpretation of the results. BV wrote the first draft of the manuscript. All authors contributed to manuscript revision, read, and approved the submitted version.

## Funding

Research of Alzheimer Center Amsterdam is part of the neurodegeneration research program of Amsterdam Neuroscience. Alzheimer Center Amsterdam is supported by Stichting Alzheimer Nederland and Stichting VUmc fonds. The chair of WF is supported by the Pasman stichting. WF is recipient of a grant by Stichting Equilibrio and of ZonMW-Memorabel funded #733050814. The SCIENCe project is supported by a research grant of stichting Dioraphte. BV is appointed on an Amsterdam Cardiovascular Sciences (ACSPhD2019P003) and an Alzheimer Nederland grant (WE.03-2017-12). FB is supported by the NIHR biomedical research centre at UCLH. MN is supported by a personal ZONMW-VICI grant 2020 (09150182010020).

## Conflict of Interest

CT received grants from the European Commission, the Dutch Research Council (ZonMW), Association of Frontotemporal Dementia/Alzheimer’s Drug Discovery Foundation, The Weston Brain Institute, Alzheimer Netherlands. CT has a collaboration contract with ADx Neurosciences, performed contract research or received grants from Probiodrug, Biogen, Esai, Toyama, Janssen prevention center, Boehringer, AxonNeurosciences, Fujirebio, EIP farma, PeopleBio, and Roche. FB is a consultant for Biogen-Idec, Bayer-Schering, Merck-Serono, Roche, Combinostics and IXICO; has received sponsorship from European Commission–Horizon 2020, National Institute for Health Research–University College London Hospitals Biomedical Research Centre, Novartis, and Merck; and serves on the editorial boards of Radiology, Neuroradiology, Multiple Sclerosis Journal, and Neurology. PS has received consultancy/speaker fees from Lilly, GE Healthcare, Novartis, Sanofi, Nutricia, Probiodrug, Biogen, Roche, Avraham, and EIP Pharma. PS has acquired grant support from GE Healthcare, Danone Research, Piramal, and MERCK. All funding was paid to the institution. MN is part of the Scientific Advisory Board of Caelus Health, The Netherlands and Kaleido Biosciences, USA. However, none of these are directly relevant to the current paper. WF received research funding from ZonMW, NWO, EU-FP7, EU-JPND, Alzheimer Nederland, CardioVascular Onderzoek Nederland, Health~Holland, Topsector Life Sciences & Health, stichting Dioraphte, Gieskes-Strijbis fonds, stichting Equilibrio, Pasman stichting, Biogen MA Inc, Boehringer Ingelheim, Life-MI, AVID, Roche BV, Fujifilm, Combinostics. WF holds the Pasman chair. WF is recipient of ABOARD, which is a public-private partnership receiving funding from ZonMW (#73305095007) and Health~Holland, Topsector Life Sciences & Health (PPP-allowance; #LSHM20106). She has performed contract research for Biogen MA Inc, and Boehringer Ingelheim. She has been an invited speaker at Boehringer Ingelheim, Biogen MA Inc, Danone, Eisai, WebMD Neurology (Medscape). WF is consultant to Oxford Health Policy Forum CIC, Roche, and Biogen MA Inc. WF participated in an advisory board of Biogen MA Inc and Roche. WF was associate editor of Alzheimer, Research & Therapy in 2020/2021. WF is associate editor at Brain. All funding was paid to the institution.

The remaining authors declare that the research was conducted in the absence of any commercial or financial relationships that could be construed as a potential conflict of interest.

## Publisher’s Note

All claims expressed in this article are solely those of the authors and do not necessarily represent those of their affiliated organizations, or those of the publisher, the editors and the reviewers. Any product that may be evaluated in this article, or claim that may be made by its manufacturer, is not guaranteed or endorsed by the publisher.
