# Supplementary material for: Gut Microbiota Composition Is Related to AD Pathology
Source: Front Immunol. 2022 Jan 31;12:794519. doi: 10.3389/fimmu.2021.794519 (PMC8843078; doi:10.3389/fimmu.2021.794519)
Supplement: Supplementary file 1 [file DataSheet_1.pdf]

## Supplement 1: Bioinformatic pipeline

16S rRNA primers were removed from the sequencing reads using seqtk (v. 1.3). The reads were subsequently processed using dada2 (v.1.18) as follows. After examining read quality profiles, 50 bases were trimmed from the 5' end of the forward reads, and 60 bases from the 5' of the reverse reads, respectively. The reads were truncated at the first base with a Q score lower than 4, then quality filtered using 2 maximum expected error for the forward reads and 4 maximum expected errors for the reverse reads, allowing for no ambiguous bases. The filtered reads were used to learn the error rates and to infer Amplicon Sequence Variants (ASVs) separately for the forward and the reverse reads. Forward and reverse ASVs were merged allowing no mismatching bases and requiring a minimum overlap of 20 bases. ASVs shorter than 350 bp, longer than 500bp, and chimeric ASVs were removed. An ASV table was constructed for the remaining ASVs. ASV taxonomy was then assigned using the dada2 'assignTaxonomy' function and the SILVA database (release 138) allowing up to 3 multiple species-level assignments (Quast et al., 2013; Callahan et al., 2016). The ASV table and taxonomy were integrated using the phyloseq R package (v.1.34.0). The ASV table was rarefied to 20000 counts per sample (McMurdie and Holmes, 2013). Of 175 sequenced samples, 5 had insufficient counts (<20000 counts per sample) and were excluded at the rarefaction stage.

**References**

Callahan, B. J., McMurdie, P. J., Rosen, M. J., Han, A. W., Johnson, A. J. A., and Holmes, S. P. (2016). DADA2: High-resolution sample inference from Illumina amplicon data. *Nat. Methods*. doi:10.1038/nmeth.3869.

Edgar, R. C. (2010). Search and clustering orders of magnitude faster than BLAST. *Bioinformatics*. doi:10.1093/bioinformatics/btq461.

Edgar, R. C. (2016). UNOISE2: improved error-correction for Illumina 16S and ITS amplicon sequencing. doi:10.1101/081257.

McMurdie, P. J., and Holmes, S. (2013). Phyloseq: An R Package for Reproducible Interactive Analysis and Graphics of Microbiome Census Data. *PLoS One*. doi:10.1371/journal.pone.0061217.

Quast, C., Pruesse, E., Yilmaz, P., Gerken, J., Schweer, T., Yarza, P., et al. (2013). The SILVA ribosomal RNA gene database project: Improved data processing and web-based tools. *Nucleic Acids Res.* doi:10.1093/nar/gks1219.

## Supplement 2: Machine learning design

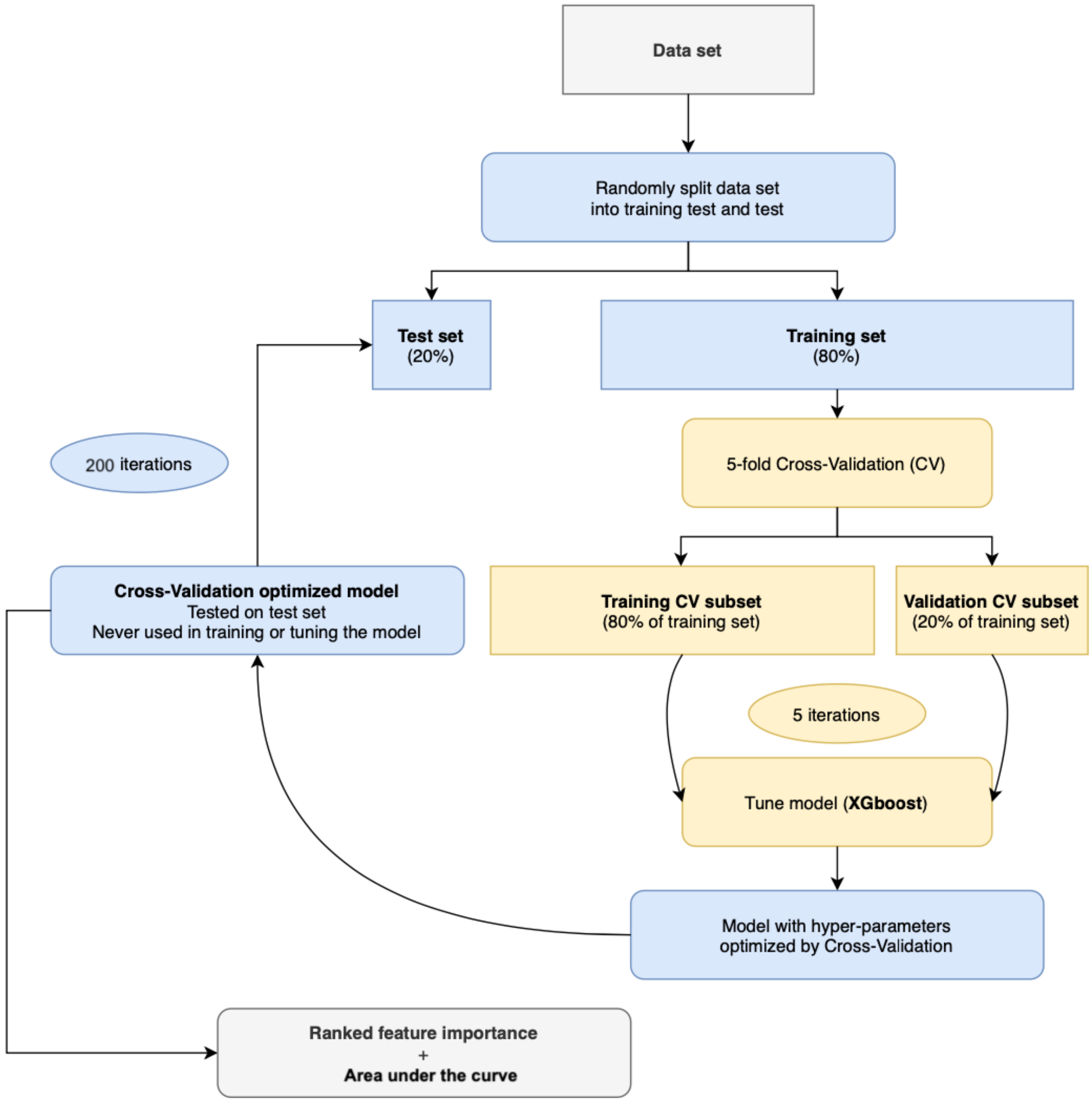


*Schematic overview of the machine learning design.*

# Supplement 3: Distribution of CSF biomarkers

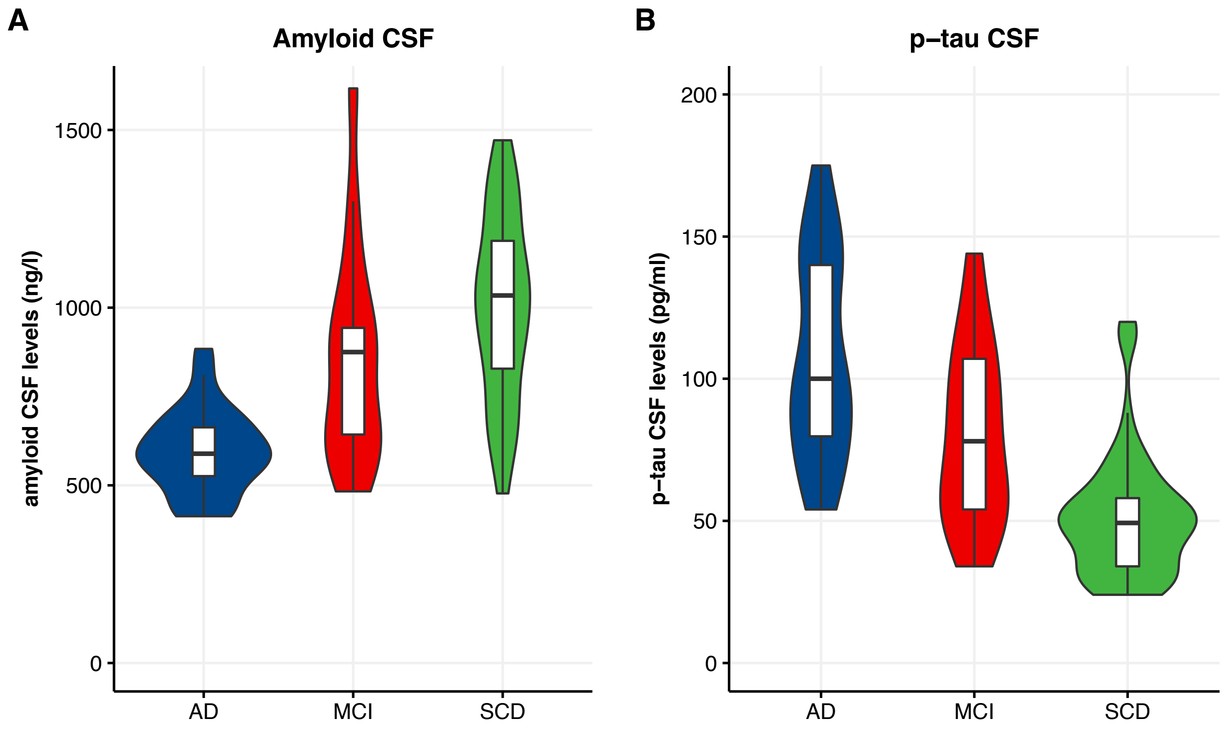


*Distribution of (A) amyloid and (B) p-tau CSF levels per diagnosis group.*

# Supplement 4: Machine learning results amyloid and p-tau CSF model

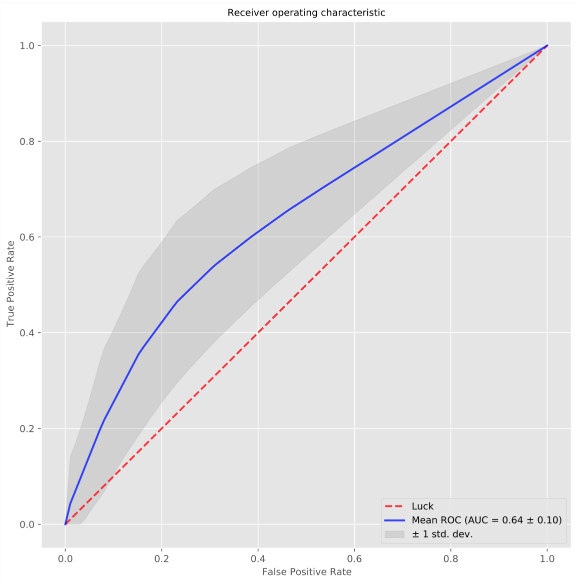


*Figure S3.1: AUC for machine learning model predicting amyloid CSF status from microbiota composition.*

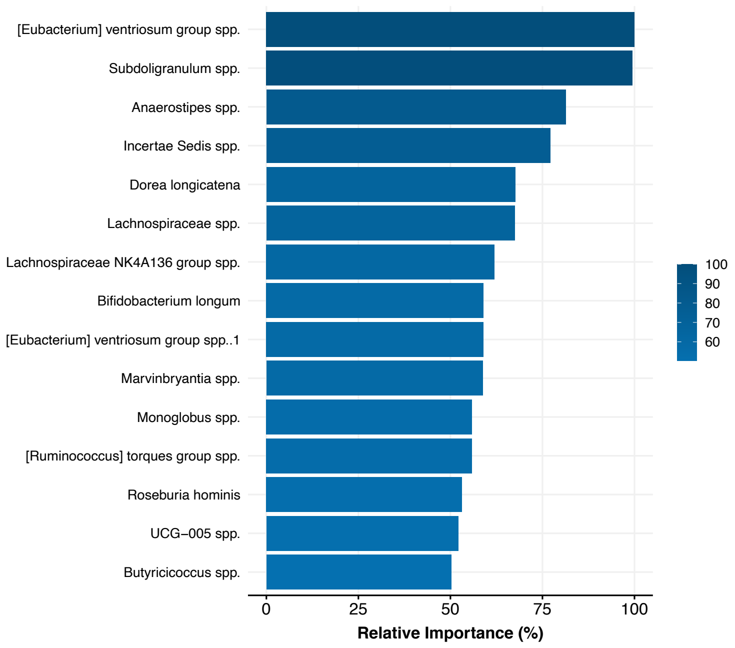


*Figure S3.2: Feature importance of highest ranked predictors of the machine learning model for amyloid CSF status.*

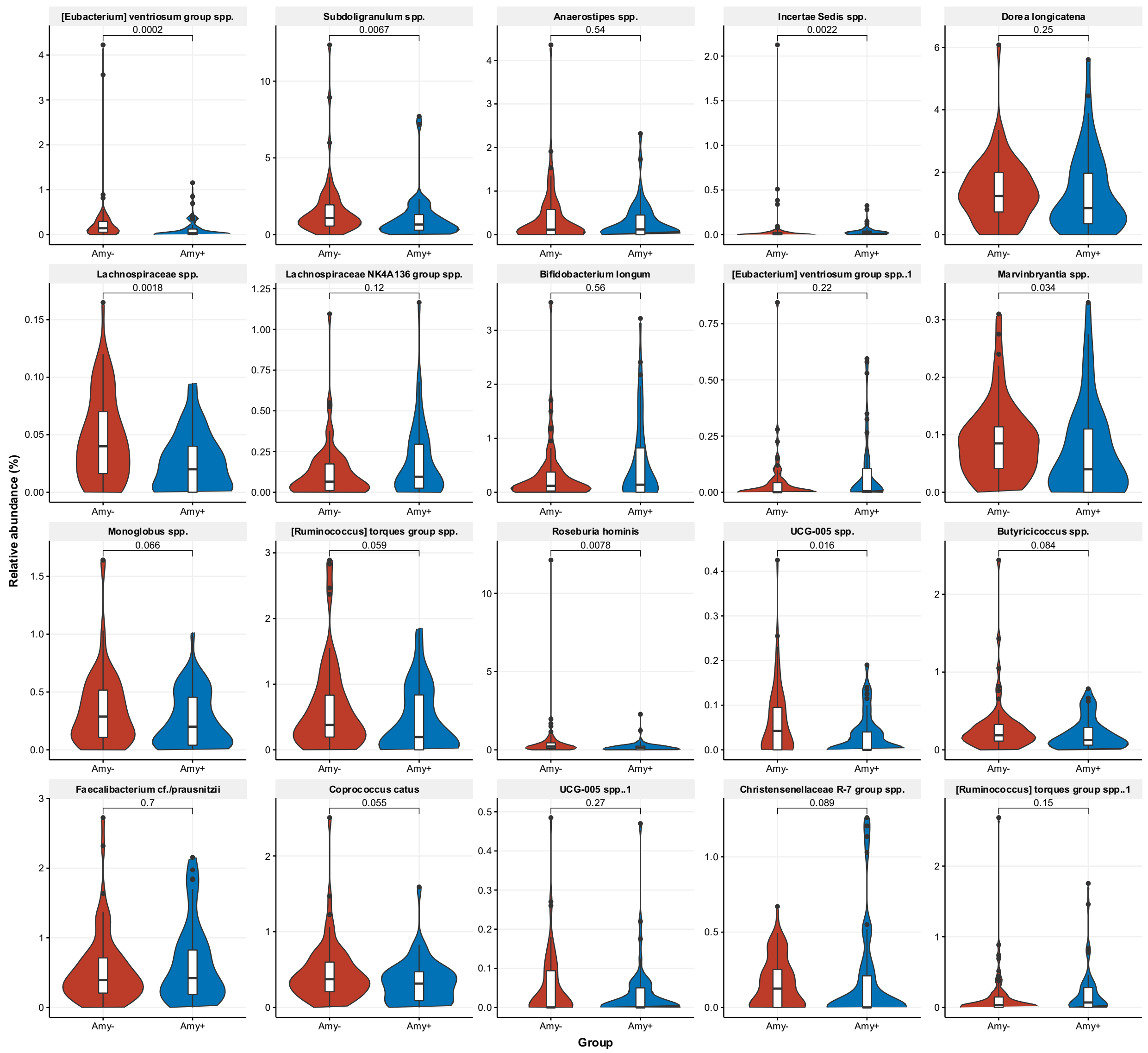


*Figure S3.3: Violin plots with differences in 20 highest ranked predictors between amyloid positive (Amy+) and amyloid negative (Amy-) subjects. Differences between groups were tested with Mann-Whitney U tests.*

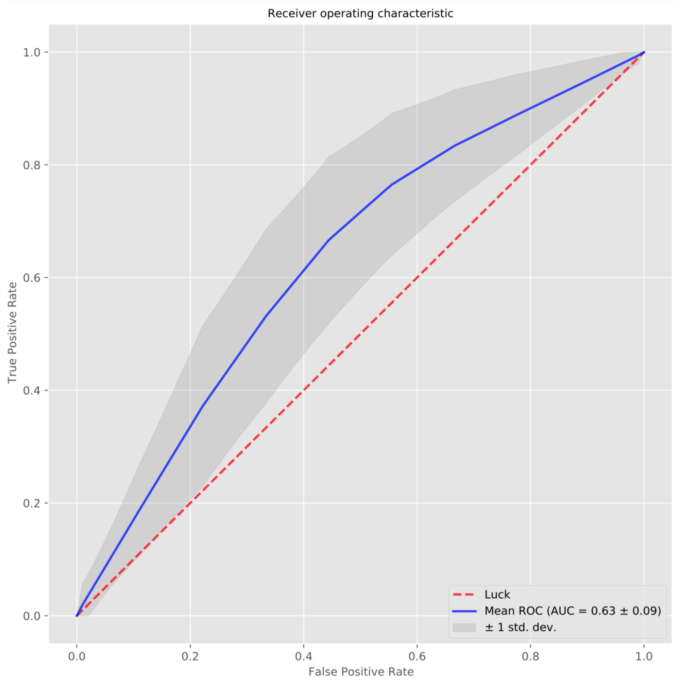


*Figure S3.4: AUC for machine learning model predicting p-tau CSF status from microbiota composition.*

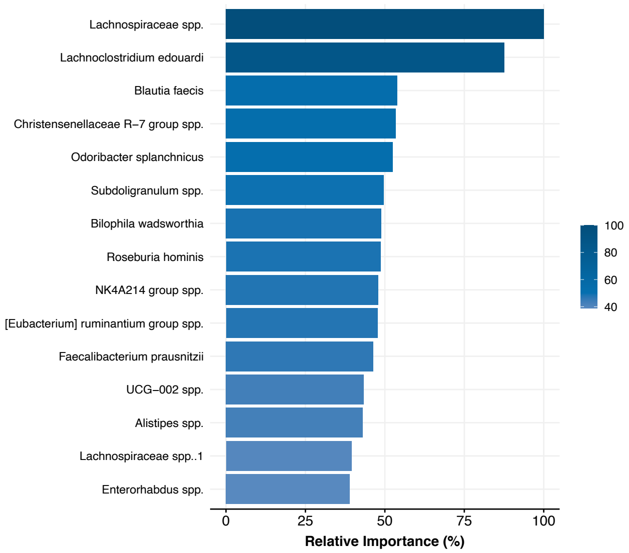


*Figure S3.5: Feature importance of highest ranked predictors of the machine learning model for p-tau CSF status.*

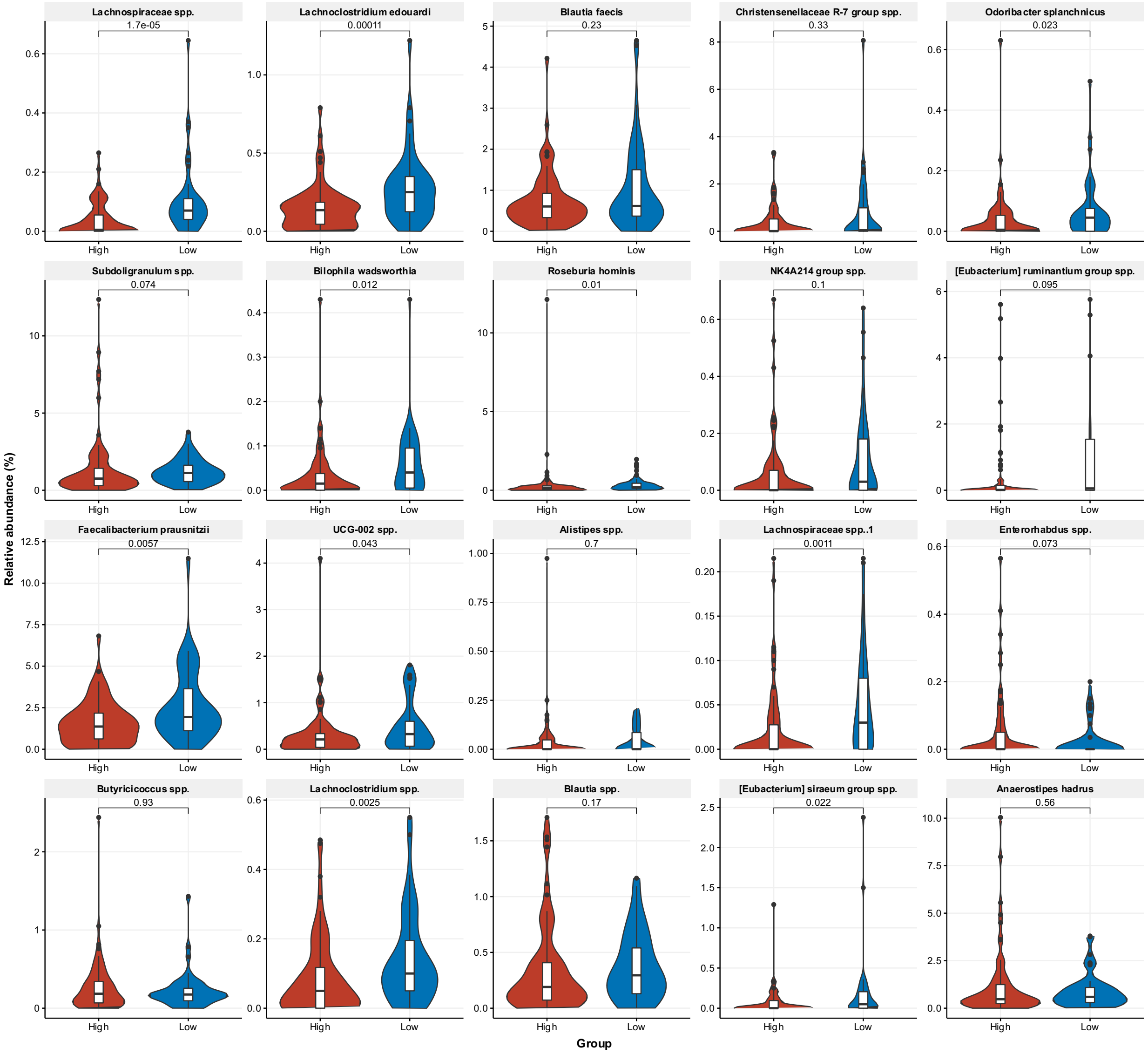


*Figure S3.6: Violin plots with differences in 20 highest ranked predictors between high and low p-tau subjects. Differences between groups were tested with Mann-Whitney U tests.*
